# Cancer Three-Dimensional Spheroids Mimic In Vivo Tumor Features, Displaying “Inner” Extracellular Vesicles and Vasculogenic Mimicry

**DOI:** 10.3390/ijms231911782

**Published:** 2022-10-04

**Authors:** Ilaria Giusti, Giuseppina Poppa, Sandra D’Ascenzo, Letizia Esposito, Anna Rita Vitale, Giuseppe Calvisi, Vincenza Dolo

**Affiliations:** 1 Department of Life, Health and Environmental Sciences, University of L’Aquila, 67100 L’Aquila, Italy; 2 Pathology Unit, San Salvatore Hospital, Via Lorenzo Natali, 1, Coppito, 67100 L’Aquila, Italy

**Keywords:** extracellular vesicles, ovarian cancer, spheroids, vasculogenic mimicry

## Abstract

The role of extracellular vesicles (EVs) as mediators of cell-to-cell communication in cancer progression is widely recognized. In vitro studies are routinely performed on 2D culture models, but recent studies suggest that 3D cultures could represent a more valid model. Human ovarian cancer cells CABA I were cultured by the hanging drop method to form tumor spheroids, that were moved to low adhesion supports to observe their morphology by Scanning Electron Microscopy (SEM) and to isolate the EVs. EVs release was verified by SEM and their identity confirmed by morphology (Transmission Electron Microscopy, TEM), size distribution (Nanoparticles Tracking Analysis), and markers (CD63, CD9, TSG-101, Calnexin). CABA I form spheroids with a clinically relevant size, above 400 μm; they release EVs on their external surface and also trap “inner” EVs. They also produce vasculogenic mimicry-like tubules, that bulge from the spheroid and are composed of a hollow lumen delimited by tumor cells. CABA I can be grown as multicellular spheroids to easily isolate EVs. The presence of features typical of in vivo tumors (inner entrapped EVs and vasculogenic mimicry) suggests their use as faithful experimental models to screen therapeutic drugs targeting these pro-tumorigenic processes.

## 1. Introduction

The term “extracellular vesicles” (EVs) refers to a group of heterogeneous spherical particles surrounded by a phospholipid bilayer; depending on their biogenesis and dimensions, the EVs are commonly classified as exosomes or small EVs (30–150 nm), microvesicles or large EVs (100–1000 nm) and apoptotic bodies (50–5000 nm) [1]. Exosomes are formed from the endolysosomal pathways because of the inward budding of the plasma membrane to form early endosomes; the subsequent maturation and the invagination of the endosomal membrane leads to the formation of multivesicular bodies which lastly, by fusing with the cell membrane, release into extracellular space the small vesicles that constitute exosomes. Microvesicles, in contrast, are directly released by the outward budding of the plasma membrane. Finally, apoptotic bodies are formed from a cytoskeletal rearrangement induced by apoptosis [1,2]. Exosomes and microvesicles have long been known to be specifically involved in intercellular communication. Although EVs were initially thought to be cellular waste material, it has been demonstrated that, depending on their composition and cellular derivation, the cells can use EVs as a regulatory mechanism for several physiological cellular processes, such as immune response [3,4,5], myelin biogenesis [6] and melanogenesis [7], cellular waste management [8], neuroprotection [9] and homeostasis in general [10,11,12]. Nowadays, the main role ascribed to EVs concerns intercellular communication, since they are released from parental cells and interact with target cells by several mechanisms such as contact with surface receptors or by releasing their cargo via fusion; this function is carried out in both physiological and pathological processes, including cancer [13,14].

Tumor-derived extracellular vesicles (tEVs) are involved in the multiple processes which ultimately sustain tumor progression and dissemination [15,16] such as: (i) induction of angiogenesis, since the formation of vessels de novo is an important mechanism for tumor growth and sustenance [17,18]; (ii) evasion and/or suppression of the immune response [19]; (iii) promotion of invasion and metastasis as the tEVs, leaving the primary tumor, can move through biological fluids and enhance a supportive environment for the tumor in other sites of the organism, inducing the formation of pre-metastatic niches and of metastases [20]; (iv) modulation of the tumor microenvironment, where tEVs can stimulate a pro-tumorigenic phenotype and behavior on normal cells [21]. Due to their multiple implications in cancer, EVs are considered potential tools not only to understand the underlying mechanisms of cancer, but also for clinical approaches in terms of early diagnosis, prognosis, and development of targeted therapies.

The 2D model (monolayer) is the routine in vitro method to culture cells and isolate EVs; this choice is mainly supported by its simplicity, reproducibility, and low costs. Nonetheless, several studies have suggested that the 2D model fails to accurately mimic the architecture and features of three-dimensional (3D) in vivo solid tumors. This is the reason why, in recent years, alternative 3D culture models are being developed. In cancer research, 3D models are considered as an intermediate model between 2D cultures and in vivo experiments, being able to represent more truthfully some characteristics of in vivo tumors, in terms of cell-to-cell and cell-to-extracellular matrix (ECM) contacts, cellular layered assembling, hypoxia, and gradients of nutrient, oxygen, and pH [22,23,24].

Several 3D models are currently available; in some of them cells are grown on exogenous 3D structures (scaffold-based models) and in some others they are not (scaffold-free models) [24,25]. In the scaffold-free cultures, cells produce and deposit their own ECM similarly to what happens in vivo and grow as aggregates named “multicellular tumor spheroids” or, simply, “spheroids”. Spheroids seem to be able to reproduce quite faithfully some structural and biological features of tumors: in addition to ECM deposition, which allows the cell-cell and ECM-cell interactions to happen, they also have a necrotic center and peripheral layers of senescent and proliferating cells (from center to periphery), gradients of oxygen, nutrients, and pH (decreasing from periphery to center), and a peculiar growth kinetic (an exponential growth followed by a plateau) [22,25,26].

Spheroids can be generated by several techniques [27], including the growth of cell suspension as a “hanging drop”: the surface tension and gravitational force allow the cells to form homogenous and multicellular spheroid at the concavity of the drop. The cells, that initially generate loose aggregates, after a few days form closer contacts by N-cadherin-E-cadherin interactions generating compact structures [26,27,28].

Along with several advantages of this method (adjustable size of a spheroid modifying the cell number, no requirement of professional expensive equipment, possibility to generate a huge number of homogeneous spheroids, reproducible size) [23,27], some issues have also been reported with this method, such as difficulty in drug addition or media replacement [24].

As media replacement is mandatory for EVs collection, only a few studies concerning EVs rely on 3D models, and still no protocols have been standardized for EVs isolation from 3D cultures.

Since it has been observed in vivo that ovarian cancers often tend to grow in ascites as cell clusters (spheroids) having the potential to metastasize [29,30,31], the spheroid model could be particularly relevant to study, in vitro, the ovarian cancer biology and how EVs contribute to cancer progression, taking advantage of an approach that is more consistent with in vivo situation. Indeed, several studies on ovarian cancer have already suggested that the culture model choice can impact, for example, sensitivity to chemotherapeutic agents [24,32].

The aim of this study was to set up an easy protocol to isolate EVs from tumor cells grown as 3D multicellular spheroids, generating a cell model that better mimics the tumor architecture in terms of cell-to-cell and cell-to-extracellular matrix contacts, hypoxia, and gradients and that can be used as an intermediate model between 2D culture models and in vivo experiments in the study of EV biology.

We found that human ovarian cancer cells CABA I can be grown as multicellular spheroids and that the implementation of a simple protocol allows easy isolation of the EVs for subsequent studies. This culture model traps “inner” EVs similarly to in vivo tissues and produces tube-like structures typical of vasculogenic mimicry, suggesting it could be useful as an experimental model closely resembling in vivo tumors.

## 2. Results

### 2.1. CABA I Cells Generate Compact Spheroids

**Figure 1:**
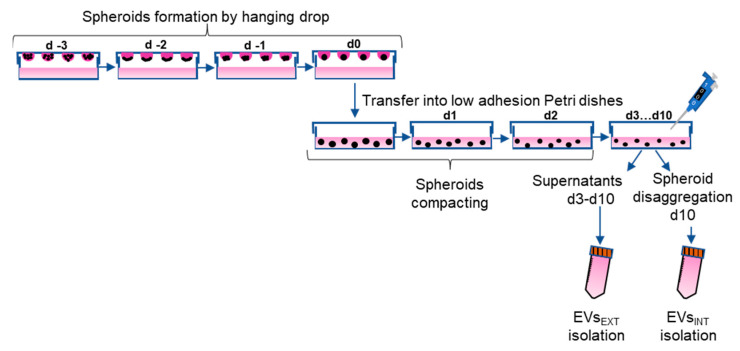
Experimental scheme. Spheroids were formed by “hanging drop” from day −3 to day 0 (d −3–d0) then transferred into low adhesion Petri dishes on d0. Spheroids were left to compact for 72 h (d0–d3) and from d3 to d10 EVs_EXT_ were collected. On d10, spheroids were disaggregated as described in Methods and EVs_INT_ were isolated. “Day 0” was chosen to signify the day spheroids were moved from drop to Petri; from day zero (d0) the previous 3 days (as d −1, d −2, d −3) and the following 10 (d1–d10) were considered.

The scheme followed to isolate EVs from CABA I cells cultured by the “hanging drop” is reported in Figure 1; EVs were isolated both from supernatants collected from day 3 to day 10 (d3-d10) (EVs_EXT_) and following the spheroids’ disaggregation on d10 (EVs_INT_).

**Figure 2:**
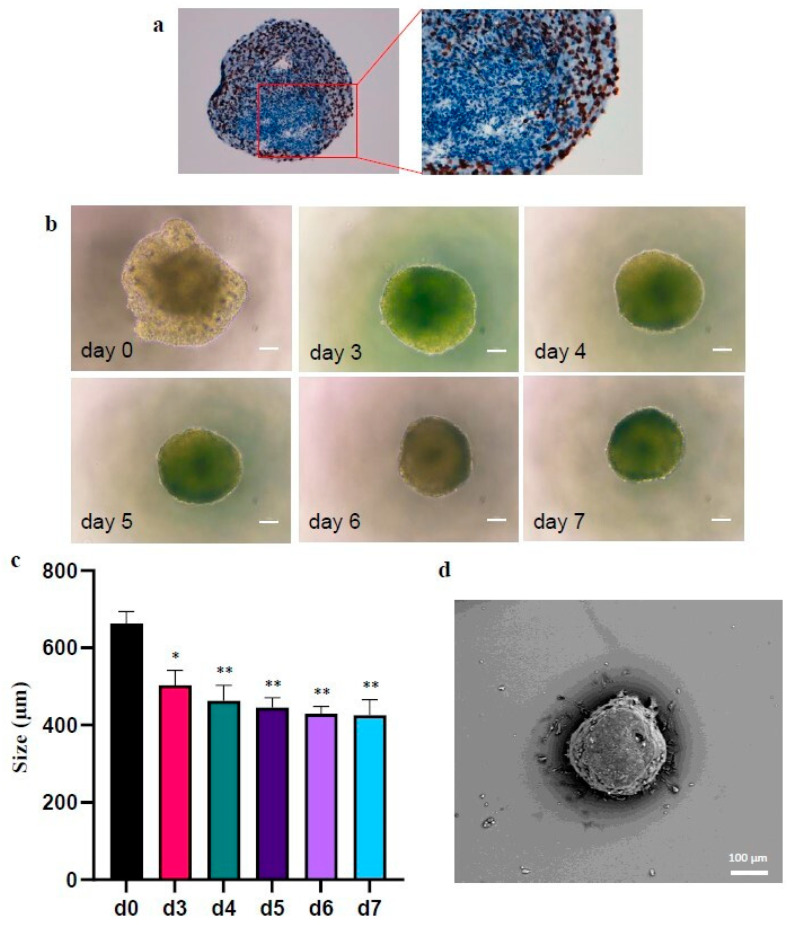
Morphology and size of a CABA I representative spheroid. (**a**) Ki67 staining of a representative spheroid (conventional immunohistochemical staining procedure). 20× magnification for the left image, 40× magnification for the right image; (**b**) Images of a representative CABA I spheroid at d0, d3–d7 from the transfer onto low attachment surface; the size bar is 100 µm. (**c**) Graph reporting the mean size (mean of two repeated measures of diameter) of the representative spheroid at days 0–7 (mean ± SD) (* *p* < 0.05; ** *p* < 0.01). (**d**) SEM image of a representative CABA I spheroid; the size bar is 100 µm.

When cultured according to the scheme in Figure 1, CABA I cells generated multicellular spheroids (Figure 2). These spheroids are a compact bunch of cells, which tend to proliferate in the peripheral layers, but not in the center, as suggested by the Ki67 staining, that stains brownish the proliferating cells’ nuclei (Figure 2a).

On day 0, the spheroid periphery cells were still not compacted but in about 72 h the spheroid became compact and further compacted in the following days (Figure 2b); in fact, the average size of spheroid diameter went from 662.3 ± 31.9 µm on d0, to 502.9 ± 38.5 µm on d3, 462.6 ± 40.3 µm on d4, 444.9 ± 25.5 µm on d5, 428.9 ± 18.9 µm on d6, 425.3 ± 40.5 µm on d7 (Figure 2c); from d3 to d7, the average size was significantly decreased compared to d0. When observed with Scanning Electron Microscopy, the compact and spherical shape of the spheroid was confirmed (Figure 2d).

**Figure 3:**
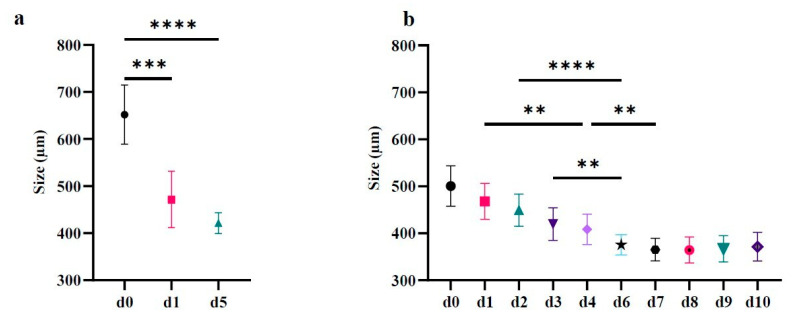
Size trend over days of CABA I spheroids. (**a**) Mean ± SD of 14 spheroids observed on d0, d1 and d5. (**b**) Mean ± SD of 35 spheroids observed on d0-d10. Kruskal–Wallis test followed by Dunn’s post-hoc test (** *p* < 0.01; ***: *p* < 0.005; **** *p* < 0.001). In each graph, asterisks on horizontal bars represents the statistical significance between the two values selected. In **b**, all values, starting from d3 are statistically significant compared to d0 (**** *p* < 0.001; only d3 ** *p* < 0.01).

The trend to compacting has been confirmed in a population of 14 and 35 spheroids (Figure 3a,b, respectively).

Figure 3a shows that the mean size significantly decreases in d1 and d5 compared to d0 (651 ± 63 µm at d0, 471.6 ± 59.9 µm at d1, 421.4 ± 22.2 µm at d5).

To better define the kinetics of compaction, a representative population of 35 spheroids was observed from d0 to d10. Figure 3b displays their mean size, highlighting that there is a significantly decrease in their diameter every 72 h. It also highlights that, between d0 and d7, every 24 h the spheroids compact by about 2–6.5% compared to the previous day. On days d7 to d10 the size remains essentially constant. Minimum, maximum, and mean size are reported in Table 1.

### 2.2. CABA I Spheroids Release EVs and Contain “Inner” EVs

**Figure 4:**
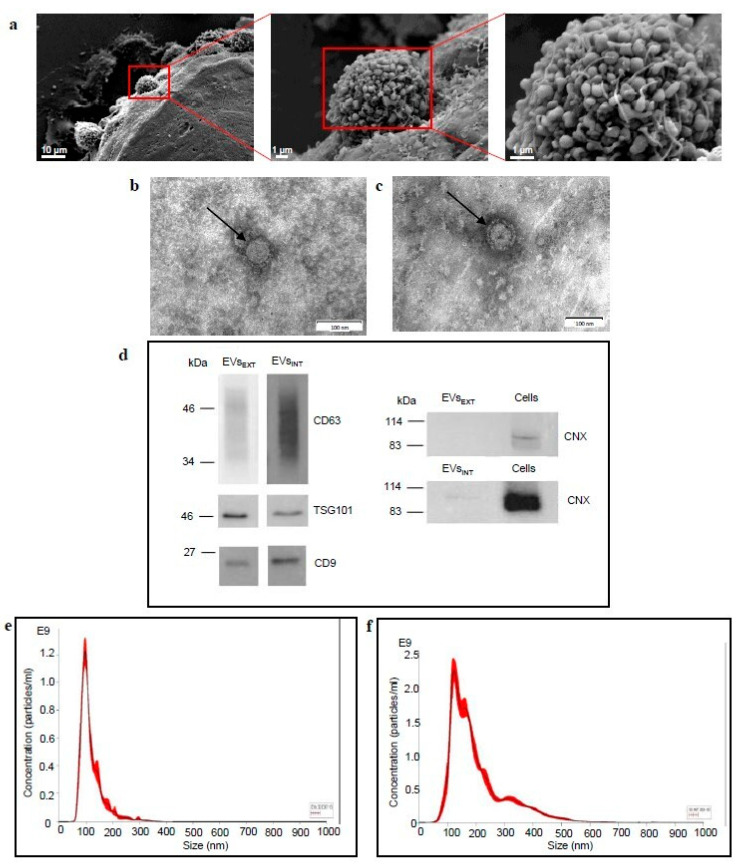
Spheroids release of EVs_EXT_ and EVs_INT_. (**a**) SEM images, representative of several observations, showing EVs release from the spheroid surface. Each red rectangle encloses the microscope field shown, in higher magnification, on the image on the right. The lower magnification on the left shows the outer surface of spheroid; higher magnifications highlight the release of EVs from cell surface. Size bar is 10 µm in the image on the left, 1 µm on other images. (**b**) Ultrastructural TEM image of EV_EXT_; the size bar is 100 nm. (**c**) Ultrastructural TEM image of EV_INT_; the size bar is 100 nm. In **b** and **c** the arrows point to EVs. (**d**) Western blots of CD63, TSG101, CD9 and calnexin (CNX) on EVs_EXT_ and EVs_INT_ samples. For CNX, that must be negative in EVs, total cell proteins are showed as positive control. (**e**) Representative NTA profile of EVs_EXT_. (**f**) Representative NTA profile of EVs_INT_.

The SEM images show the release of heterogeneous size EVs from the outer surface of a representative spheroid (Figure 4a). The TEM ultrastructural analysis confirms the presence of intact and rounded EVs, enclosed in a lipidic bilayer (appearing as a thin white filament), both released in the supernatant (Figure 4b) and entrapped inside the spheroids (Figure 4c); the evaluation by Western blot of positive (CD63, TSG101, CD9) and negative (calnexin, CNX) markers specific for EVs identification further confirms that the samples isolated by ultracentrifugation consist of EVs (Figure 4d). The size distribution assayed by nanoparticle tracking analysis (NTA) highlights that EVs_EXT_ are rather homogeneous in size, and mainly of mean size <200 nm (particles <200 nm: 95, 59 ± 1.26 %); normalized on 100 spheroids, the mean release of particles in EVs_EXT_ sample is 1.57 × 10^9^ ± 4.92 × 10^8^ (Figure 4e). EVs_INT_ sample, instead, contains particles more heterogeneous in size; (particles <200 nm: 80.57 ± 10.51 %); normalized on 100 spheroids, the mean release of particles in EVs_INT_ sample is 1.76 × 10^9^ ± 3.26 × 10^8^ (Figure 4f).

**Figure 5:**
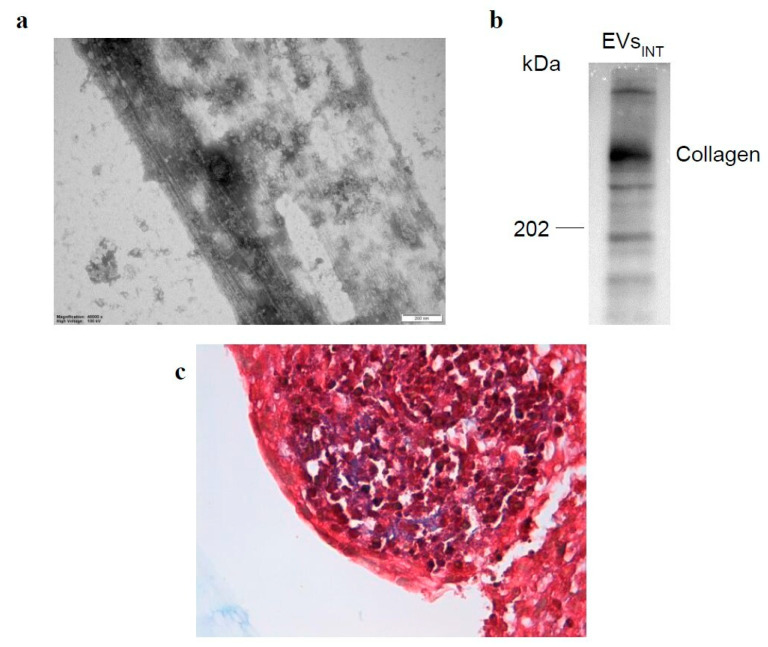
Collagen in EVs_INT_ sample. (**a**) Ultrastructural TEM image of EVINT sample highlighting the presence of fibers; the size bar is 200 nm. (**b**) Western blotting of collagen in EVs_INT_ sample. (**c**) Masson trichrome stain (conventional staining procedure) most likely showing, in blue/purple, the presence of connective tissue; image 60×.

The TEM analysis highlighted that EVs_INT_ can be surrounded by long fibers (Figure 5a) that are part of the extracellular matrix necessary to compact the spheroid and which we believe to be, at least partly, collagen as confirmed by the Western blot of EVs_INT_ samples; indeed, the samples of EVs_INT_ revealed the presence of several fragments of collagen ranging in size between 170–290 kDa (Figure 5b); the Masson trichrome stain, too, most likely shows the presence of connective tissue (Figure 5c).

### 2.3. CABA I Spheroids Exhibit a Vasculogenic Mimicry-like Process

**Figure 6:**
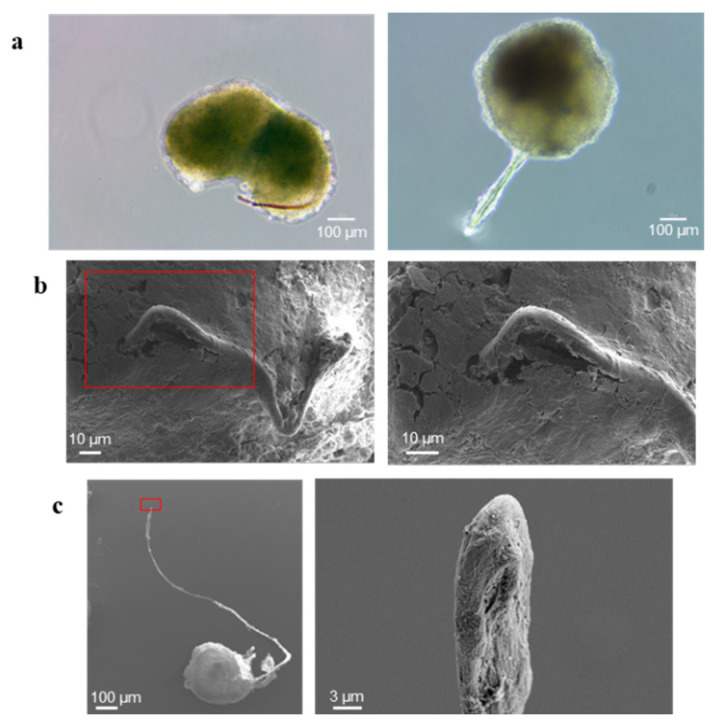
Vasculogenic mimicry-like phenomenon. (**a**) Images of some representative spheroids containing tubules observed by optical microscopy; the size bar is 100 µm. (**b**,**c**) SEM images of representative spheroids presenting tubules: (**b**) shows the tubule exiting from the spheroid; (**c**) shows the tip of the tubule; each red rectangle encloses the microscope field shown, in higher magnification, on the image on the right, highlighting the cavity of the tubule. The size bar is 10 µm in (**b**), 100 and 3 µm in (**c**), in order from the left to the right image.

The presence of long prolongations bulging out from CABA I spheroids was frequently observed (Figure 6): in each batch at least from 3–8% of spheroids exhibit these prolongations. In some cases, they appear intensely colored pink (Figure 6a, left image), suggesting that they contain medium and, therefore, are hollow within. The analysis by SEM confirmed that the spheroids can generate these prolongations, which come out from the mass of the spheroid (Figure 6b) and whose tip could be closed, as in the one showed as representative (Figure 6c).

**Figure 7:**
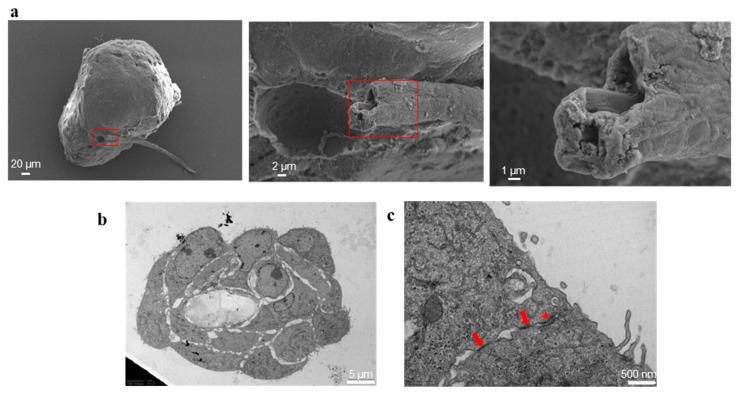
Lumen inside the vasculogenic mimicry-like tubules. (**a**): SEM images highlighting the tubule lumen; each red rectangle encloses the microscope field shown, in higher magnification, on the image on the right, highlighting the cavity of the tubule. The size bar is 20, 2 and 1 µm, in order from the left to the right image. (**b**): TEM image of a sagittal section of the tubule, highlighting the hollow lumen delimited by cells. The size bar is 5 µm. (**c**): representative TEM image showing the cellular junctions: the arrows point to tight junctions, the arrowhead points to desmosome. The size bar is 500 nm.

SEM observation not only confirmed that these protrusions are hollow inside (Figure 7a), but also indicated that these tubule-like structures are defined by cellular elements as further highlighted by TEM. In fact, TEM observation of a sagittal section of tubule shows that its central lumen is hollow and enclosed by cells (Figure 7b); cells delimiting the lumen are held together by tight junctions, which can be identified as dense lines, and desmosomes, which can be identified as dense plaques with converging filaments on their cytoplasmic side (Figure 7c).

## 3. Discussion

Ovarian cancer (OC) is one of the most frequently diagnosed tumors and a leading cause of tumor death in women [24,33]; since specific symptoms are lacking and effective early screening is not available, the diagnosis is often late and most commonly occurs when most women already are in an advanced stage and have abdominal metastases and ascites [34,35,36]. This lowers the 5-years survival rate, and most of the deaths occur within 2 years from diagnosis [35].

In OC, similarly to what happens for other tumors, the tumor growth and progression depend on interactions between cancer cells and their microenvironment, and EVs have been widely demonstrated to be mediators of such intercellular communication: EV signaling, in vivo and in vitro, modulates the proliferation and motility/invasiveness of tumor cells, the angiogenesis, the immune response, the activation of fibroblasts in cancer-associated fibroblasts, and the pre-metastatic niche formation [21,37,38,39,40,41,42,43].

Even if EV study has often relied on 2D culture models, emerging evidence suggests that 3D approaches represent a more reliable model to study EVs biology in OC, in order to fully understand their contribution to tumor progression and improve clinical translational outcomes [24].

Nevertheless, studies focused on EVs conducted on 3D models are very few and a protocol for the isolation of EVs from 3D models has not yet been standardized [44]. Here we propose a protocol to easily isolate EVs from human OC cells grown as spheroids and demonstrate for the first time that this culture model not only allows the collection of EVs released into the culture medium, but also contains entrapped “inner” EVs.

The CABA I spheroids were generated by the “hanging drop” method, optimizing the number of cells and the culture time in the drop, as described. Subsequently, to proceed with the isolation of the EVs, the spheroids were moved to supports coated with an anti-adhesion solution; this solution allowed to cultivate the spheroids for further 10 days, preventing their adhesion on the plate, as well as the adhesion of any cells detached from the spheroids. The daily change of medium, performed to collect EVs, did not affect the low-adhesion surface generated by the coating solution.

The generated spheroids are compact multicellular 3D structures, with an outer proliferative layer of cells, and not proliferating cells in the inner mass, as described in the literature [45].

At d0 the dimensions of the spheroids were greater than in the following days, in which progressive compaction occurred. Although in the various preparations at d0 the spheroids’ dimensions were found to be variable (from 530.1 ± 37.1 µM up to 744.5 ± 73.7 µM), as their compaction proceeded, they tended to become more uniform: on d4-d5 the mean size was between 408.43 ± 32.3 µM and 445 ± 18.9 µM; this could suggest that, although the cells can aggregate in a more or less loose way when in the drop, once compaction has started, the final dimensions reached are substantially dictated by the number of cells contained in the spheroid.

To verify if this culture model could allow for EVs isolation, their release was first evaluated and confirmed by SEM observation. Thus, EVs released into the culture medium from d3 to d10 were collected as described; it is to note that, on the days when EVs were collected (d3–d10), a large part of the spheroids had a clinically relevant size: an estimated diameter of about 400 μm should ensure the generation of spheroids that mimic some features of in vivo solid tumor, such as the formation of a necrotic core and gradients generation for nutrients and oxygen (the diffusion for them both is about 100 μm in depth) [24,28,46].

We wondered if, similarly to what has been described for human tumor tissues [47,48], spheroids could trap “inner” EVs. So, on d10 spheroids were disaggregated by hydrolyzing the ECM deposited by cells, confirming that some EVs are entrapped inside the multicellular spheroid. The identity of these “inner” EVs (EVs_INT_), as well as that of EVs_EXT_, has been fully confirmed as suggested by the International Society for Extracellular Vesicles (ISEV) [49] by means of TEM observation, NTA, and markers analysis.

The NTA population analysis of the EVs_INT_ sample showed a remarkable dimensional heterogeneity in this sample if compared to the EVs_EXT_ sample; given that it is obtained following the digestion of collagen deposited by the cells, we hypothesized that it could contain collagen fragments. We confirmed this hypothesis both by TEM observations, that highlight the presence of fibers in the sample, and by Western blot analysis, which shows that collagen fragments of different sizes are contained in the sample. This suggests optimizing the digestion of the ECM to obtain a pure population of EVs_INT_ before proceeding with further studies.

Despite this, to the best of our knowledge, this is the first time it has been shown that in vitro 3D culture models may contain “inner” EVs entrapped within; it is possible to hypothesize that these EVs, being released from a more prohibitive environment (in terms of hypoxia and low nutrient levels), could have different molecular and functional characteristics. It will be necessary, therefore, to deepen this aspect to understand whether it is mandatory to isolate those “inner” EVs to have a more representative EV population of the whole tumor mass.

Finally, we have observed that quite often spheroids produce some extensions; generally, they are found singularly, but occasionally we have also observed more than one per spheroid (data not shown). Although, commonly, these prolongations appeared translucent, on a few occasions they had an intense pink color, as if they contained some culture medium. We, therefore, hypothesized that they could be tube-like structures, generated by a vasculogenic mimicry process. This is a process whereby cancer cells organize themselves into vascular-like structures to catch oxygen and nutrients, in order to become independent from endothelial cells’ presence, assuring supplies by normal blood vessels or angiogenesis [50].

SEM observation of the spheroids confirmed that these prolongations come out from the internal mass of the spheroid and that, indeed, they are hollow inside. TEM observation further shows that these prolongations consist of a hollow space delimited by few tumor cells held together by tight junctions and desmosomes. The tip, on the contrary, is closed; this evidence led us to think that the observed protrusions are formed with a mechanism similar to that with which new vessels are generated during angiogenesis, that is with an endothelial cell converting into a “tip cell” that precedes and paves the way for the endothelial cells behind, that follow the tip as a “stalk” and then organize themselves in a hollow structure that will constitute the new vessel lumen [51,52].

It has been observed that vasculogenic mimicry is usually associated with poor prognosis and survival in cancer, being related to high tumor grade and increased ability in progression, invasion, and metastasis [53,54]. Hence, molecular pathways responsible for vasculogenic mimicry are considered as promising novel therapeutic targets in anti-cancer therapy [55].

It is not the first time vasculogenic mimicry has been observed in human ovarian cancer [56,57,58,59] but, as far as we know, this is the first time it is observed that multicellular spheroids produce hollow tube-like structures. The possibility for human ovarian cancer spheroids to make tube-like structures by vasculogenic mimicry underlines even more how this culture model is strongly representative of what happens in in vivo settings, suggesting that 3D spheroids could bridge the gap between 2D and in vivo models and be useful, for example, in the screening for the drugs designed to inhibit vasculogenic mimicry.

Moreover, the possibility to culture these spheroids in low adhesion conditions for several days and to repeatedly replace the culture medium also suggests this culture model could be befitting when it comes to EVs isolation and study from a more representative model.

## 4. Materials and Methods

### 4.1. Cell Culture

2D culture. CABA I is a human ovarian cancer cell line established from the ascitic fluid of an ovarian carcinoma patient not undergoing drug treatment (33); CABA I cell line had been characterized by means of short tandem repeats profiling and cytogenetic analysis (34). Cells were cultured as monolayers in RPMI-1640 with 5% of FBS (Fetal Bovine Serum) heat-inactivated. The medium was also supplemented with 1× penicillin/streptomycin and 2 mM L-glutamine. CABA I cells were tested for the absence of mycoplasma and cultured at 37 °C in a humidified atmosphere, 5% CO_2_.

FBS, RPMI-1640, glutamine, penicillin, and streptomycin were all purchased from Euroclone (Euroclone SpA, Milan, Italy).

3D culture. The 3D spheroids were generated by the hanging drop method, using a cell suspension of 1,500,000 cells/mL cultured in suspended drops of 20 μL placed on the inverted lid of a 35 mm Petri dish (Euroclone SpA, Milan, Italy). To prevent the drops from drying out due to evaporation problems, RPMI was placed at the bottom of the dish. CABA I spheroids were cultured in drop form for 72 h in the same medium as the 2D cultures (from d −3 to d0; Figure 1). Afterwards, on d0 the spheroids were transferred into Petri dishes coated with the commercially available anti-adhesion solution Bio Flat Flex Coating Solution (faCellitate, Mannheim, Germany) and cultured in complete medium for further 72 h to allow for their compaction (d0–d3; Figure 1).

### 4.2. EVs Collection

For EVs isolation, the complete medium was replaced by RPMI-1640 supplemented with 5% of EVs-free FBS HyClone (GE Healthcare Life Sciences, Boston, MA, USA), 1 mL/10 spheroids. The medium was collected from 3D culture daily for one week (d3–d10; Figure 1), centrifuged at 600× *g* for 15 min and 1500× *g* for 30 min to remove cells and large debris, respectively, and stored at 4 °C.

On d10, the spheroids were disaggregated according to a protocol modified by Crescitelli et al. (35) to collect “inner” EVs: collagenase D and DNase I (Merck Life Sciences S.r.l., Taufkirchen, Germany) were added in RPMI-1640 at the final concentration of 2 mg/mL and 40 U/mL, respectively; then the spheroids were incubated at 37 °C until complete breakage (30–45 min), pipetting them every 5 min to ensure enzymatic efficacy. The resulting cell suspension was centrifuged to remove cells and the supernatant treated as previously described, to obtain the “inner” EVs enriched supernatant.

To collect EVs, supernatants underwent ultracentrifugation (100,000× *g*, 90 min, 4 °C, Rotor 70Ti, Quick-Seal Ultra-Clear tubes, kadj 221, brake 9) in an Optima XPN-110 Ultracentrifuge (Beckman Coulter, Brea, CA, USA) (36).

Pelleted EVs were resuspended in Dulbecco’s phosphate-buffered saline (EuroClone, Milan, Italy). Bradford method was performed to quantify the protein levels associated with isolated EVs and BSA (Bovine Serum Albumin; Merck Life Sciences S.r.l., Taufkirchen, Germany) was used as standard.

### 4.3. Electron Microscopy

Scanning electron microscopy. Spheroids were explored for their structure and EVs release by scanning electron microscopy (SEM): for this purpose, they were placed on Poly-L-Lysine-coated coverslips (Merck Life Sciences S.r.l., Taufkirchen, Germany), fixed with 2% glutaraldehyde (Electron Microscopy Sciences, Hatfield, PA, USA) in PBS overnight at 4 °C, gradually dehydrated with ethanol series (30–100%) and dried through a graduated series of ethanol:HMDS mixtures (2:1 ethanol:HMDS and 1:2 ethanol:HMDS respectively), until pure HMDS (Hexamethyldisilazane, Electron Microscopy Sciences, Hatfield, PA, USA); finally, samples were glued onto stubs, chromium-coated in a Q 150T ES Sputter coater (Quorum Technologies, Laughton, UK), and detected with a Zeiss GeminiSEM 500 (Zeiss, Oberkochen, Germany).

Transmission electron microscopy. The ultrastructure of isolated EVs and of tubule was analyzed by transmission electron microscopy (TEM).

As for EVs, after being properly diluted, the samples were placed on 300 mesh carbon-coated copper grids (Electron Microscopy Sciences, Hatfield, PA, USA) in a humidified chamber at room temperature for 15 min; then they were fixed in 2% glutaraldehyde in PBS for 10 min and rinsed with Milli-Q water three times, each one for 3 min. Finally, negative staining was performed using a 2% solution of pH 7 phosphotungstic acid.

As for tubules, a spheroid was fixed in a 2% Gluteraldehyde solution. The sample was then washed firstly in PBS and then in 0.1 M Sodium Cacodylate buffer; a post-fixation with 1% Osmium Oxide and a staining with 1% Uranyl Acetate were performed. The sample was then dehydrated in a graded scale of ethanol (30–100%), embedded with resin and polymerized in an oven at 60 °C for approximately 72 h. Ultrathin sections of the tubule were cut with a Reichert-Jung Ultracut E ultramicrotome, stained with 20% uranyl acetate and lead citrate solution and observed by TEM.

Grids were examined with a Philips CM 100 TEM 80 kV transmission electron microscope and the images were captured by a Kodak digital camera.

### 4.4. Nanoparticle Tracking Analysis

Particles contained in EVs samples were analyzed, in terms of concentration and size, by nanoparticle tracking analysis (NTA), using a NanoSight NS300 (NanoSight Ltd., Amesbury, UK). Briefly, EV-enriched pellets were resuspended in sterile, filtered PBS to generate a dilution in which 20–120 particles/frame were tracked and, for each sample, 5 recordings of 60 s (camera level 15–16) were performed examining 1498 frames in total, that were captured and analyzed by applying optimized settings. Data were analyzed with the NTA software, which provided the concentration measurements (particles/mL) and size distribution profiles for the particles in the solution.

### 4.5. Western Blot

For Western blots, 8 µg of EVs and 30 µg of cell extracts were resolved by 10% sodium dodecyl sulfate-polyacrylamide gel electrophoresis (SDS-PAGE) under different conditions, depending on the primary antibody used (non-reducing conditions and with heating for CD63; reducing conditions and with heating for both CD9 and TSG101; non-reducing conditions and without heating for CANX and collagen I) and transferred to nitrocellulose membranes (GE Healthcare Life Sciences, Boston, MA, USA) Non-specific binding sites were blocked for 90 min in 10% non-fat dry milk in TBS containing 0.5% Tween-20 (TBS-T), under agitation at RT. The nitrocellulose membranes were then incubated at 4 °C ON with the following primary antibodies: mouse monoclonal anti-CD63 (dilution 1:400; sc-59286), mouse monoclonal anti-CD9 (dilution 1:400; sc-13118) (both from Santa Cruz Biotechnology, Inc., Dallas, Texas, TX, USA)), rabbit polyclonal anti-TSG101 (dilution 1:2000; ab83881), rabbit polyclonal anti-CANX (dilution 1:1000; ab81541) and rabbit polyclonal Anti-collagen I (dilution 1:1000; ab-34710) (Immunological Sciences, Rome, Italy).

After several washes in TBS-T, the membranes were incubated with an appropriate peroxidase-conjugated secondary antibody: goat anti-mouse IgG-HRP (dilution 1:10000; sc-2005), or goat anti-rabbit IgG-HRP (dilution 1:7500; sc-2004) (both Santa Cruz Biotechnology Inc. Dallas, TX, USA) for 1 h. All antibodies were diluted in TBS-T containing 1% non-fat dry milk.

Finally, after washing in TBS-T, the reactive bands on the membranes were detected and acquired as images with the documentation system on gel Alliance LD2 (UVItec, Cambridge, UK), using a chemiluminescence detection kit (SuperSignal West Pico Chemiluminescent Substrate; Merck Life Sciences S.r.l., Taufkirchen, Germany).

### 4.6. Statistical Analysis

Data shown are from at least three independent experiments and are presented as mean ± SD. Statistical significance was determined using the Kruskal–Wallis test followed by Dunn’s test. Calculations were performed using GraphPad Prism 4 software (GraphPad, San Diego, CA, USA); results were considered statistically significant when *p* < 0.05 (*), *p* < 0.01 (**), *p* < 0.005 (***), *p* < 0.001 (****).

## 5. Conclusions

Human ovarian cancer cells can be grown as multicellular spheroids to easily isolate EVs. This culture model traps “inner” EVs similarly to in vivo tissues and produce tube-like structures typical of vasculogenic mimicry. This approach could be useful as an experimental model closing resembling in vivo tumors.

## Figures and Tables

**Table 1:** Minimum, maximum and mean size of 34 spheroids population for each day.

	Minimum Size (µM)	Maximum Size (µM)	Mean Size (µM)
d0	400.0 ± 21.2	573.2 ± 19.8	500.6 ± 43.0
d1	402.2 ± 34.5	522.0 ± 20.9	468.1 ± 38.3
d2	383.4 ± 23.7	509.1 ± 39.9	449.5 ± 34.2
d3	370.1 ± 42.6	494.6 ± 31.5	419.6 ± 34.7
d4	347.1 ± 39.4	481.9 ± 30	408.4 ± 32.3
d6	331.4 ± 22.6	425.3 ± 7.4	375.8 ± 21.7
d7	324.0 ± 24.6	427.5 ± 8.0	365.4 ± 23.8
d8	313.4 ± 22.0	432.1 ± 13.2	364.5 ± 27.6
d9	312.6 ± 14.2	416.1 ± 17.5	367.1 ± 27.9
d10	311.0 ± 8.7	424.9 ± 25.8	371.6 ± 30.6

d stands for day.

## Data Availability

The datasets generated during and/or analyzed during the current study are available from the corresponding author on reasonable request.

## Abbreviations

2D	two-dimensional
3D	three-dimensional
BSA	Bovine Serum Albumin
CD63	Cluster of differentiation 63
CD9	Cluster of differentiation 9
CNX	Calnexin
ECM	Extracellular matrix
EVs	Extracellular vesicles
FBS	Foetal Bovine Serum
HMDS	Hexamethyldisilazane
HRP	Horseradish Peroxidase
NTA	Nanoparticles Tracking Assay
OC	Ovarian Cancer
PBS	Phosphate Buffered Saline
SEM	Scanning Electron Microscopy
TEM	Transmission Electron Microscopy
tEVs	Tumor-derived extracellular vesicles
TSG101	Tumor susceptibility gene 101

## References

[1] Doyle L., Wang M. (2019). Overview of Extracellular Vesicles, Their Origin, Composition, Purpose, and Methods for Exosome Isolation and Analysis. Cells.

[2] Teng F., Fussenegger M. (2021). Shedding Light on Extracellular Vesicle Biogenesis and Bioengineering. Adv. Sci..

[3] Weiss R., Gröger M., Rauscher S., Fendl B., Eichhorn T., Fischer M.B., Spittler A., Weber V. (2018). Differential Interaction of Platelet-Derived Extracellular Vesicles with Leukocyte Subsets in Human Whole Blood. Sci. Rep..

[4] Hovhannisyan L., Czechowska E., Gutowska-Owsiak D. (2021). The Role of Non-Immune Cell-Derived Extracellular Vesicles in Allergy. Front. Immunol..

[5] Robbins P.D., Morelli A.E. (2014). Regulation of Immune Responses by Extracellular Vesicles. Nat. Rev. Immunol..

[6] Bakhti M., Winter C., Simons M. (2011). Inhibition of Myelin Membrane Sheath Formation by Oligodendrocyte-Derived Exosome-like Vesicles. J. Biol. Chem..

[7] Kim N.-H., Choi S.-H., Kim C.-H., Lee C.H., Lee T.R., Lee A.-Y. (2014). Reduced MiR-675 in Exosome in H19 RNA-Related Melanogenesis via MITF as a Direct Target. J. Investig. Dermatol..

[8] Yuana Y., Sturk A., Nieuwland R. (2013). Extracellular Vesicles in Physiological and Pathological Conditions. Blood Rev..

[9] Frühbeis C., Fröhlich D., Kuo W.P., Amphornrat J., Thilemann S., Saab A.S., Kirchhoff F., Möbius W., Goebbels S., Nave K.-A. (2013). Neurotransmitter-Triggered Transfer of Exosomes Mediates Oligodendrocyte–Neuron Communication. PLoS Biol..

[10] Stahl P.D., Raposo G. (2019). Extracellular Vesicles: Exosomes and Microvesicles, Integrators of Homeostasis. Physiology.

[11] Yates A.G., Pink R.C., Erdbrügger U., Siljander P.R., Dellar E.R., Pantazi P., Akbar N., Cooke W.R., Vatish M., Dias-Neto E. (2022). In Sickness and in Health: The Functional Role of Extracellular Vesicles in Physiology and Pathology In Vivo: Part I: Health and Normal Physiology. J. Extracell. Vesicle.

[12] Yates A.G., Pink R.C., Erdbrügger U., Siljander P.R., Dellar E.R., Pantazi P., Akbar N., Cooke W.R., Vatish M., Dias-Neto E. (2022). In Sickness and in Health: The Functional Role of Extracellular Vesicles in Physiology and Pathology In Vivo: Part II: Pathology. J. Extracell. Vesicle.

[13] Bebelman M.P., Smit M.J., Pegtel D.M., Baglio S.R. (2018). Biogenesis and Function of Extracellular Vesicles in Cancer. Pharmacol. Ther..

[14] van Niel G., Carter D.R.F., Clayton A., Lambert D.W., Raposo G., Vader P. (2022). Challenges and Directions in Studying Cell–Cell Communication by Extracellular Vesicles. Nat. Rev. Mol. Cell Biol.

[15] Beltraminelli T., Perez C.R., De Palma M. (2021). Disentangling the Complexity of Tumor-Derived Extracellular Vesicles. Cell Rep..

[16] Shehzad A., Islam S.U., Shahzad R., Khan S., Lee Y.S. (2021). Extracellular Vesicles in Cancer Diagnostics and Therapeutics. Pharmacol. Ther..

[17] Huang M., Liu M., Huang D., Ma Y., Ye G., Wen Q., Li Y., Deng L., Qi Q., Liu T. (2022). Tumor Perivascular Cell-Derived Extracellular Vesicles Promote Angiogenesis via the Gas6/Axl Pathway. Cancer Lett..

[18] Giannandrea D., Platonova N., Colombo M., Mazzola M., Citro V., Adami R., Maltoni F., Ancona S., Dolo V., Giusti I. (2022). Extracellular Vesicles Mediate the Communication between Multiple Myeloma and Bone Marrow Microenvironment in a NOTCH Dependent Way. Haematologica.

[19] Marar C., Starich B., Wirtz D. (2021). Extracellular Vesicles in Immunomodulation and Tumor Progression. Nat. Immunol..

[20] Wang S.E. (2020). Extracellular Vesicles and Metastasis. Cold Spring Harb. Perspect. Med..

[21] Giusti I., Di Francesco M., Poppa G., Esposito L., D’Ascenzo S., Dolo V. (2022). Tumor-Derived Extracellular Vesicles Activate Normal Human Fibroblasts to a Cancer-Associated Fibroblast-Like Phenotype, Sustaining a Pro-Tumorigenic Microenvironment. Front. Oncol..

[22] Barbosa M.A.G., Xavier C.P.R., Pereira R.F., Petrikaitė V., Vasconcelos M.H. (2021). 3D Cell Culture Models as Recapitulators of the Tumor Microenvironment for the Screening of Anti-Cancer Drugs. Cancers.

[23] Nunes A.S., Barros A.S., Costa E.C., Moreira A.F., Correia I.J. (2019). 3D Tumor Spheroids as in Vitro Models to Mimic in Vivo Human Solid Tumors Resistance to Therapeutic Drugs. Biotechnol. Bioeng..

[24] Yee C., Dickson K.-A., Muntasir M.N., Ma Y., Marsh D.J. (2022). Three-Dimensional Modelling of Ovarian Cancer: From Cell Lines to Organoids for Discovery and Personalized Medicine. Front. Bioeng. Biotechnol..

[25] Knight E., Przyborski S. (2015). Advances in 3D Cell Culture Technologies Enabling Tissue-like Structures to Be Created In Vitro. J. Anat..

[26] Costa E.C., Moreira A.F., de Melo-Diogo D., Gaspar V.M., Carvalho M.P., Correia I.J. (2016). 3D Tumor Spheroids: An Overview on the Tools and Techniques Used for Their Analysis. Biotechnol. Adv..

[27] Ryu N.-E., Lee S.-H., Park H. (2019). Spheroid Culture System Methods and Applications for Mesenchymal Stem Cells. Cells.

[28] Han S.J., Kwon S., Kim K.S. (2021). Challenges of Applying Multicellular Tumor Spheroids in Preclinical Phase. Cancer Cell Int..

[29] Raghavan S., Ward M.R., Rowley K.R., Wold R.M., Takayama S., Buckanovich R.J., Mehta G. (2015). Formation of Stable Small Cell Number Three-Dimensional Ovarian Cancer Spheroids Using Hanging Drop Arrays for Preclinical Drug Sensitivity Assays. Gynecol. Oncol..

[30] Boylan K.L.M., Manion R.D., Shah H., Skubitz K.M., Skubitz A.P.N. (2020). Inhibition of Ovarian Cancer Cell Spheroid Formation by Synthetic Peptides Derived from Nectin-4. Int. J. Mol. Sci..

[31] Singh M.S., Goldsmith M., Thakur K., Chatterjee S., Landesman-Milo D., Levy T., Kunz-Schughart L.A., Barenholz Y., Peer D. (2020). An Ovarian Spheroid Based Tumor Model That Represents Vascularized Tumors and Enables the Investigation of Nanomedicine Therapeutics. Nanoscale.

[32] Myungjin Lee J., Mhawech-Fauceglia P., Lee N., Cristina Parsanian L., Gail Lin Y., Andrew Gayther S., Lawrenson K. (2013). A Three-Dimensional Microenvironment Alters Protein Expression and Chemosensitivity of Epithelial Ovarian Cancer Cells in Vitro. Lab. Investig..

[33] Berek J.S., Renz M., Kehoe S., Kumar L., Friedlander M. (2021). Cancer of the Ovary, Fallopian Tube, and Peritoneum: 2021 Update. Int. J. Gynecol. Obstet..

[34] Pereira M., Matuszewska K., Jamieson C., Petrik J. (2021). Characterizing Endocrine Status, Tumor Hypoxia and Immunogenicity for Therapy Success in Epithelial Ovarian Cancer. Front. Endocrinol..

[35] Croft P.K., Sharma S., Godbole N., Rice G.E., Salomon C. (2021). Ovarian-Cancer-Associated Extracellular Vesicles: Microenvironmental Regulation and Potential Clinical Applications. Cells.

[36] Bray F., Ferlay J., Soerjomataram I., Siegel R.L., Torre L.A., Jemal A. (2018). Global Cancer Statistics 2018: GLOBOCAN Estimates of Incidence and Mortality Worldwide for 36 Cancers in 185 Countries. CA A Cancer J. Clin..

[37] Alharbi M., Lai A., Guanzon D., Palma C., Zuñiga F., Perrin L., He Y., Hooper J.D., Salomon C. (2019). Ovarian Cancer-Derived Exosomes Promote Tumour Metastasis *in Vivo*: An Effect Modulated by the Invasiveness Capacity of Their Originating Cells. Clin. Sci..

[38] Giusti I., Di Francesco M., D’ Ascenzo S., Palmerini M.G., Macchiarelli G., Carta G., Dolo V. (2018). Ovarian Cancer-Derived Extracellular Vesicles Affect Normal Human Fibroblast Behavior. Cancer Biol. Ther..

[39] Kelleher R.J., Balu-Iyer S., Loyall J., Sacca A.J., Shenoy G.N., Peng P., Iyer V., Fathallah A.M., Berenson C.S., Wallace P.K. (2015). Extracellular Vesicles Present in Human Ovarian Tumor Microenvironments Induce a Phosphatidylserine-Dependent Arrest in the T-Cell Signaling Cascade. Cancer Immunol. Res..

[40] Labani-Motlagh A., Israelsson P., Ottander U., Lundin E., Nagaev I., Nagaeva O., Dehlin E., Baranov V., Mincheva-Nilsson L. (2016). Differential Expression of Ligands for NKG2D and DNAM-1 Receptors by Epithelial Ovarian Cancer-Derived Exosomes and Its Influence on NK Cell Cytotoxicity. Tumor Biol..

[41] Czystowska-Kuzmicz M., Sosnowska A., Nowis D., Ramji K., Szajnik M., Chlebowska-Tuz J., Wolinska E., Gaj P., Grazul M., Pilch Z. (2019). Small Extracellular Vesicles Containing Arginase-1 Suppress T-Cell Responses and Promote Tumor Growth in Ovarian Carcinoma. Nat. Commun..

[42] Yokoi A., Yoshioka Y., Yamamoto Y., Ishikawa M., Ikeda S., Kato T., Kiyono T., Takeshita F., Kajiyama H., Kikkawa F. (2017). Malignant Extracellular Vesicles Carrying MMP1 MRNA Facilitate Peritoneal Dissemination in Ovarian Cancer. Nat. Commun..

[43] Cai J., Gong L., Li G., Guo J., Yi X., Wang Z. (2021). Exosomes in Ovarian Cancer Ascites Promote Epithelial–Mesenchymal Transition of Ovarian Cancer Cells by Delivery of MiR-6780b-5p. Cell Death Dis..

[44] Eguchi T., Sheta M., Fujii M., Calderwood S.K. (2022). Cancer Extracellular Vesicles, Tumoroid Models, and Tumor Microenvironment. Semin. Cancer Biol..

[45] Zanoni M., Cortesi M., Zamagni A., Arienti C., Pignatta S., Tesei A. (2020). Modeling Neoplastic Disease with Spheroids and Organoids. J. Hematol. Oncol..

[46] Heredia-Soto V., Redondo A., Berjón A., Miguel-Martín M., Díaz E., Crespo R., Hernández A., Yébenes L., Gallego A., Feliu J. (2018). High-Throughput 3-Dimensional Culture of Epithelial Ovarian Cancer Cells as Preclinical Model of Disease. Oncotarget.

[47] Crescitelli R., Lässer C., Lötvall J. (2021). Isolation and Characterization of Extracellular Vesicle Subpopulations from Tissues. Nat. Protoc..

[48] Li S.-R., Man Q.-W., Gao X., Lin H., Wang J., Su F.-C., Wang H.-Q., Bu L.-L., Liu B., Chen G. (2021). Tissue-Derived Extracellular Vesicles in Cancers and Non-Cancer Diseases: Present and Future. J. Extracell. Vesicles.

[49] Théry C., Witwer K.W., Aikawa E., Alcaraz M.J., Anderson J.D., Andriantsitohaina R., Antoniou A., Arab T., Archer F., Atkin-Smith G.K. (2018). Minimal Information for Studies of Extracellular Vesicles 2018 (MISEV2018): A Position Statement of the International Society for Extracellular Vesicles and Update of the MISEV2014 Guidelines. J. Extracell. Vesicles.

[50] Fernández-Cortés M., Delgado-Bellido D., Oliver F.J. (2019). Vasculogenic Mimicry: Become an Endothelial Cell “But Not So Much”. Front. Oncol..

[51] Zeng A., Wang S.-R., He Y.-X., Yan Y., Zhang Y. (2021). Progress in Understanding of the Stalk and Tip Cells Formation Involvement in Angiogenesis Mechanisms. Tissue Cell.

[52] Dallinga M.G., Boas S.E., Klaassen I., Merks R.H., van Noorden C.J., Schlingemann R.O. (2015). Tip Cells in Angiogenesis. eLS.

[53] Luo Q., Wang J., Zhao W., Peng Z., Liu X., Li B., Zhang H., Shan B., Zhang C., Duan C. (2020). Vasculogenic Mimicry in Carcinogenesis and Clinical Applications. J. Hematol. Oncol..

[54] Wei X., Chen Y., Jiang X., Peng M., Liu Y., Mo Y., Ren D., Hua Y., Yu B., Zhou Y. (2021). Mechanisms of Vasculogenic Mimicry in Hypoxic Tumor Microenvironments. Mol. Cancer.

[55] Ge H., Luo H. (2018). Overview of Advances in Vasculogenic Mimicry—A Potential Target for Tumor Therapy. CMAR.

[56] Racordon D., Valdivia A., Mingo G., Erices R., Aravena R., Santoro F., Bravo M.L., Ramirez C., Gonzalez P., Sandoval A. (2017). Structural and Functional Identification of Vasculogenic Mimicry In Vitro. Sci. Rep..

[57] Tang J., Wang J., Fan L., Li X., Liu N., Luo W., Wang J., Wang Y., Wang Y. (2016). CRGD Inhibits Vasculogenic Mimicry Formation by Down-Regulating UPA Expression and Reducing EMT in Ovarian Cancer. Oncotarget.

[58] Millimaggi D., Mari M., D’Ascenzo S., Giusti I., Pavan A., Dolo V. (2009). Vasculogenic Mimicry of Human Ovarian Cancer Cells: Role of CD147. Int. J. Oncol..

[59] Ayala-Domínguez L., Olmedo-Nieva L., Muñoz-Bello J.O., Contreras-Paredes A., Manzo-Merino J., Martínez-Ramírez I., Lizano M. (2019). Mechanisms of Vasculogenic Mimicry in Ovarian Cancer. Front. Oncol..

